# A Novel Three-Dimensional Imaging System Based on Polysaccharide Staining for Accurate Histopathological Diagnosis of Inflammatory Bowel Diseases

**DOI:** 10.1016/j.jcmgh.2022.07.001

**Published:** 2022-07-12

**Authors:** Satoshi Nojima, Shoichi Ishida, Kei Terayama, Katsuhiko Matsumoto, Takahiro Matsui, Shinichiro Tahara, Kenji Ohshima, Hiroki Kiyokawa, Kansuke Kido, Koto Ukon, Shota Y. Yoshida, Tomoki T. Mitani, Yuichiro Doki, Tsunekazu Mizushima, Yasushi Okuno, Etsuo A. Susaki, Hiroki R. Ueda, Eiichi Morii

**Affiliations:** 1 Department of Pathology, Osaka University Graduate School of Medicine, Osaka, Japan; 2 Department of Immunopathology, WPI Immunology Frontier Research Center, Osaka University, Osaka, Japan; 3 Graduate School of Medical Life Science, Yokohama City University, Kanagawa, Japan; 4 Department of Biomedical Data Intelligence, Graduate School of Medicine, Kyoto University, Kyoto, Japan; 5 Laboratory for Synthetic Biology, RIKEN Center for Biosystems Dynamics Research, Osaka, Japan; 6 Department of Gastroenterological Surgery, Osaka University Graduate School of Medicine, Osaka, Japan; 7 Department of Therapeutics for Inflammatory Bowel Diseases, Osaka University Graduate School of Medicine, Osaka, Japan; 8 Integrated Frontier Research for Medical Science Division, Institute for Open and Transdisciplinary Research Initiatives (OTRI), Osaka University, Osaka, Japan; 9 RIKEN Center for Computational Science, Hyogo, Japan; 10 Department of Biochemistry and Systems Biomedicine, Juntendo University Graduate School of Medicine, Tokyo, Japan; 11 Department of Systems Pharmacology, University of Tokyo Graduate School of Medicine, Tokyo, Japan

**Keywords:** Histopathology, 3D Imaging, Tissue Clearing, Deep Learning, Inflammatory Bowel Diseases, AUC, area under the curve, CD, Crohn’s disease, 2D, two-dimensional, 3D, three-dimensional, DLS, deep learning system, IBD, inflammatory bowel disease, NSC, non-specific colitis, PAFhy, periodic acid and fluorescein dye FAM hydrazide, PAS, periodic acid-Schiff, PBS, phosphate-buffered saline, UC, ulcerative colitis

## Abstract

**Background & Aims:**

Tissue-clearing and three-dimensional (3D) imaging techniques aid clinical histopathological evaluation; however, further methodological developments are required before use in clinical practice.

**Methods:**

We sought to develop a novel fluorescence staining method based on the classical periodic acid-Schiff stain. We further attempted to develop a 3D imaging system based on this staining method and evaluated whether the system can be used for quantitative 3D pathological evaluation and deep learning–based automatic diagnosis of inflammatory bowel diseases.

**Results:**

We successfully developed a novel periodic acid–FAM hydrazide (PAFhy) staining method for 3D imaging when combined with a tissue-clearing technique (PAFhy-3D). This strategy enabled clear and detailed imaging of the 3D architectures of crypts in human colorectal mucosa. PAFhy-3D imaging also revealed abnormal architectural changes in crypts in ulcerative colitis tissues and identified the distributions of neutrophils in cryptitis and crypt abscesses. PAFhy-3D revealed novel pathological findings including spiral staircase-like crypts specific to inflammatory bowel diseases. Quantitative analysis of crypts based on 3D morphologic changes enabled differential diagnosis of ulcerative colitis, Crohn’s disease, and non-inflammatory bowel disease; such discrimination could not be achieved by pathologists. Furthermore, a deep learning–based system using PAFhy-3D images was used to distinguish these diseases The accuracies were excellent (macro-average area under the curve = 0.94; F1 scores = 0.875 for ulcerative colitis, 0.717 for Crohn’s disease, and 0.819 for non-inflammatory bowel disease).

**Conclusions:**

PAFhy staining and PAFhy-3D imaging are promising approaches for next-generation experimental and clinical histopathology.

## Graphical abstract

**Figure:**
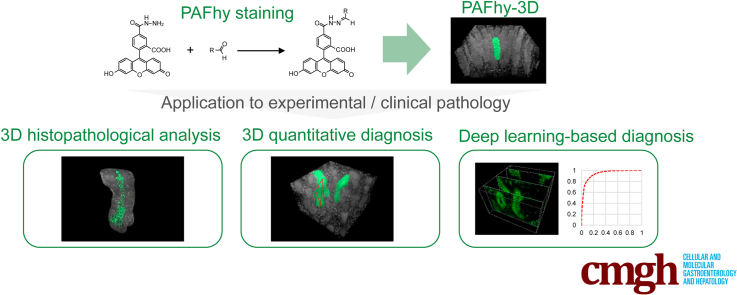

**Summary**

A novel three-dimensional imaging system based on polysaccharide staining enables detailed 3D histopathological analysis and enhances the clinicopathological diagnostic accuracies of inflammatory bowel diseases.

Pathological diagnosis contributes to the determination of final diagnosis, establishment of treatment strategy, and evaluation of treatment effects. Pathologists identify causes of disease based on morphologic changes (eg, cell atypia, degree of inflammation and fibrosis, and presence of pathogens) through the microscopic observation of patient-derived tissues. The current gold standard for pathological diagnosis involves histologic examination with H&E-stained tissues on glass slides by means of bright-field optical transmission microscopy. In addition to H&E, periodic acid-Schiff (PAS), alcian blue, and Elastica van Gieson staining methods have important roles in pathological diagnosis; they use a dye or chemical with an affinity for the particular tissue components. These staining methods enable visualization of tissue components and cells. PAS staining is used to detect substances that contain polysaccharides (eg, mucins, glycogen, glycoproteins, and glycolipids) in tissues.^1^ It was introduced by McManus^2^ in 1946 as a method to observe mucin and other structures using the Schiff reagent after treatment with periodic acid (HIO_4_). PAS staining is also useful for the detection of other polysaccharides^3^, ^4^, ^5^, ^6^, ^7^; it enables accurate diagnosis of various diseases including glomerulonephritis, fungal infection, and mucin-secreting tumors.^8^, ^9^, ^10^, ^11^ Although these methods provide information concerning morphologic changes in diseased tissue, they have some limitations. For instance, glass slide-based conventional methods provide only planar images and are unable to visualize three-dimensional (3D) anatomic structures.

Remarkable advances have been made in biological imaging. Among them, 3D imaging based on tissue-clearing reagents is a promising technique. Tissue clearing renders an organ transparent, thereby enabling the acquisition of volumetric images via confocal fluorescence microscopy, multiphoton fluorescence microscopy, and light-sheet fluorescence microscopy. Three-dimensional imaging with tissue clearing is useful for clinical histopathology^12^, ^13^, ^14^, ^15^, ^16^, ^17^, ^18^, ^19^, ^20^, ^21^, ^22^, ^23^, ^24^, ^25^, ^26^, ^27^, ^28^, ^29^, ^30^, ^31^, ^32^ and avoids the limitations of conventional histopathology. However, for clinical applications, further developments are needed in terms of staining methods, optical devices, and preparation/storage of clinical specimens.

Recent developments in artificial intelligence have been driven by breakthroughs in artificial neural networks, often termed *deep learning*.^33^ Although deep learning is useful for clinicopathological diagnosis,^34^^,^^35^ most studies thus far have used whole-slide images of H&E-stained glass slides. The acquisition of digital images from glass slides using high-resolution scanners might cause non-negligible loss of information during analog-to-digital conversion of image data. Therefore, novel digital image-based methods for data acquisition are needed for use in clinical pathology-focused deep learning.

Here, we developed a novel fluorescence staining method in combination with periodic acid and the fluorescein dye FAM hydrazide (PAFhy) staining. PAFhy staining was used for 3D imaging together with CUBIC tissue-clearing reagents (PAFhy-3D). This technique enabled the visualization of crypt architecture in inflammatory bowel disease (IBD) tissues. Quantitative evaluation of crypt morphologic changes enabled differential diagnosis of IBDs, which could not be performed by pathologists. Furthermore, the PAFhy-3D images could be used for deep learning-based diagnostics.

## Results

### Screening of Fluorescent Probes

**Figure 1:**
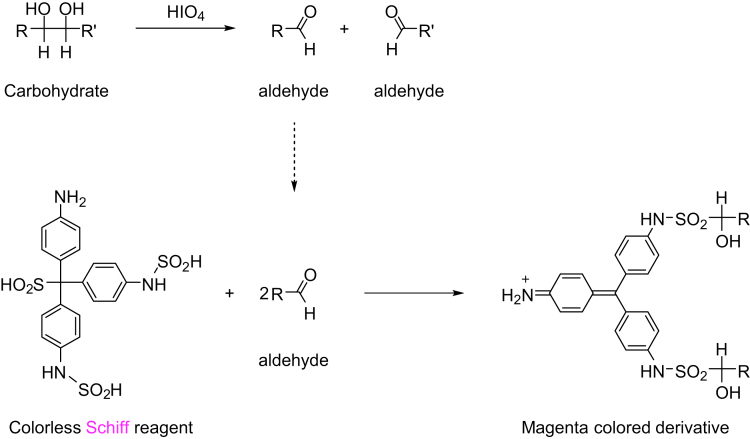
**Principle of PAS stain.** Polysaccharides react with periodic acid to produce an aldehyde, which binds to the Schiff reagent.

**Figure 2:**
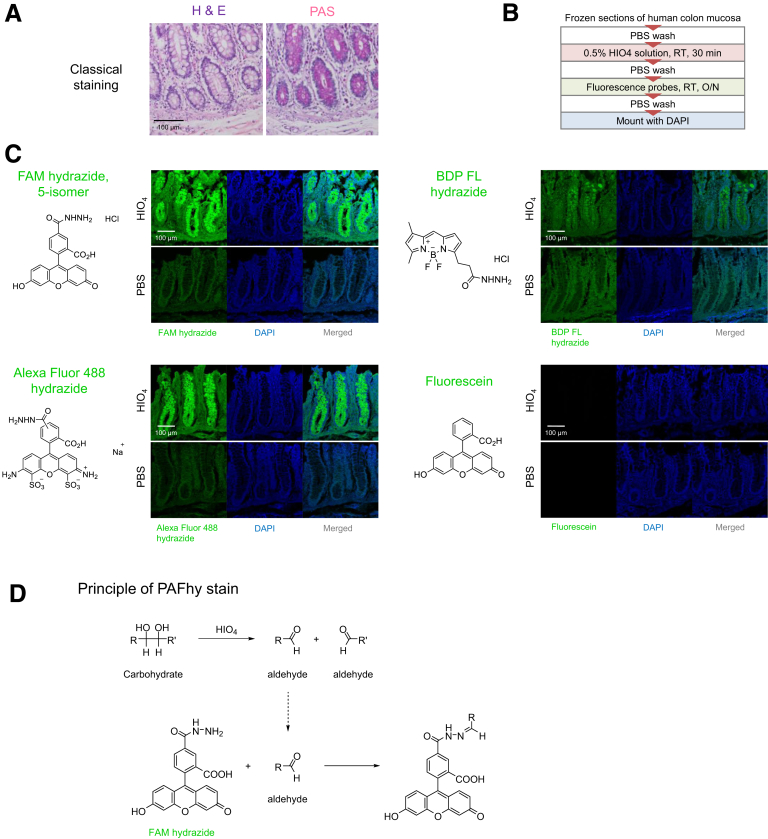
**Screening of fluorescent probes.** (*A*) H&E- or PAS-stained images of human colonic mucosa tissue used in screening. In PAS-stained images, mucus in goblet cells is *bright magenta*. Scale bar, 100 μm. (*B*) Staining procedure for frozen sections of human colonic mucosa. O/N, overnight; RT, room temperature. (*C*) Representative images of human colonic mucosae stained with fluorescent probes and corresponding structural formulas. Staining was performed with or without oxidation by periodic acid. Scale bar, 100 μm. (*D*) Principle of PAFhy staining. Polysaccharides react with periodic acid to produce an aldehyde, which binds to FAM hydrazide.

We first performed screening of fluorescence probes compatible with the Schiff reagent (Figure 1). Frozen sections of human colonic mucosa, in which PAS-positive mucus is contained in goblet cells, were used for screening (Figure 2*A*). The Schiff reagent reacts with aldehydes; thus, we screened probes with aldehyde-reactive hydrazide groups—FAM hydrazide, Alexa Fluor 488 hydrazide, BDP FL hydrazide, and fluorescein (negative control). After the application of FAM hydrazide and Alexa Fluor 488 hydrazide after HIO_4_ treatment (Figure 2*B*), mucus in goblet cells was intensely stained (Figure 2*C*). No such signal was evident if the oxidation step was omitted, suggesting that FAM hydrazide and Alexa Fluor 488 hydrazide react with aldehyde groups (Figure 2*D*). We named this the PAFhy staining method.

### Development of a Novel 3D Imaging System

**Figure 3:**
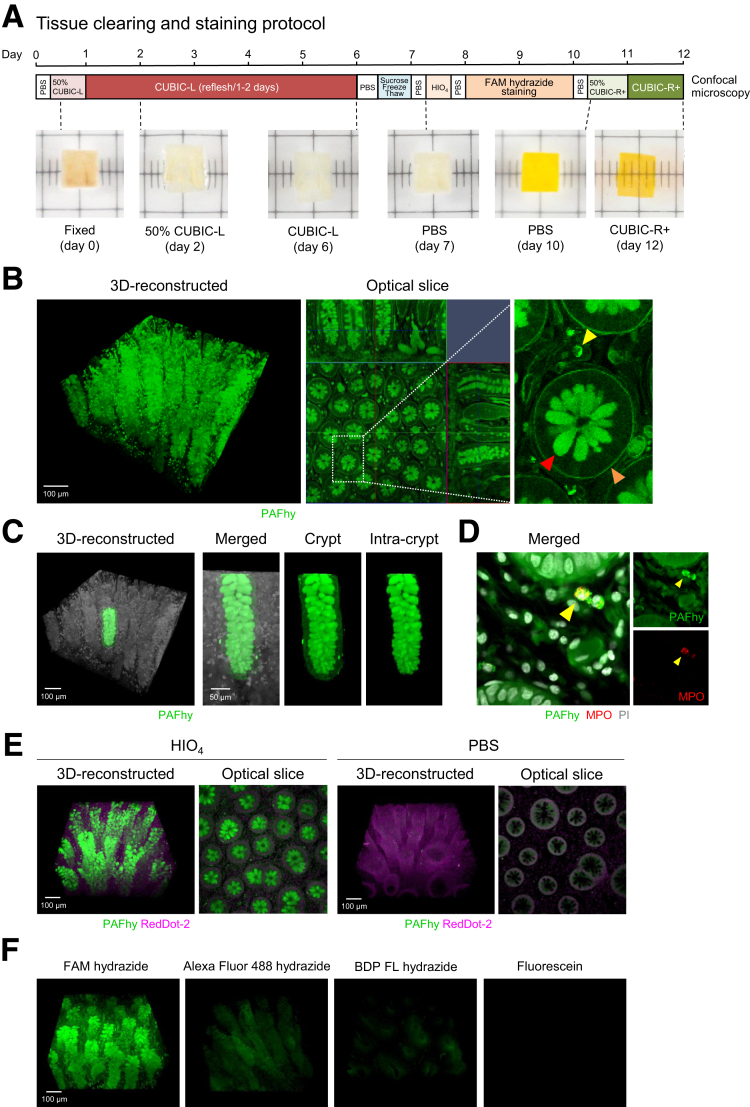
**Three-dimensional imaging of human colonic mucosae by PAFhy-3D.** (*A*) Clearing and staining of a sheet-shaped specimen of human colonic mucosa. (*B*) A representative reconstructed 3D image of the PAFhy-stained human colonic mucosa shown in (*A*). Enlarged image shows PAFhy-positive signals of mucus in goblet cells (*red arrowhead*), basement membrane (*orange arrowhead*), and granules in neutrophils (*yellow arrowhead*). Scale bar, 100 μm. (*C*) A crypt highlighted in the reconstructed 3D image in (*B*). In enlarged images, crypts with or without merge are highlighted, along with background tissue and intra-crypt mucus in goblet cells. Scale bar, 100 or 50 μm. (*D*) Image of human colonic mucosa stained with PAFhy and reacted with Alexa Fluor 647-conjugated anti-myeloperoxidase (MPO) antibody and propidium iodide (PI). Images were obtained from near the lamina propria surface of colonic mucosa by confocal microscopy. (*E*) Reconstructed 3D images and optical slices of human colonic mucosae obtained by 3D imaging with PAFhy staining with or without periodic acid oxidation. RedDot-2 was used to counterstain nuclei. Scale bar, 100 μm. (*F*) Reconstructed 3D images of human colonic mucosae stained with PAFhy (FAM hydrazide), Alexa Fluor 488 hydrazide, BDP FL hydrazide, or fluorescein. Images were obtained using the same conditions. Scale bar, 100 μm.

Next, we developed a 3D imaging system based on PAFhy staining plus tissue clearing and CUBIC 3D imaging.^36^, ^37^, ^38^ A sheet-shaped specimen of human colonic mucosa was cleared by CUBIC and subjected to PAFhy staining (Figure 3*A*). The reconstructed 3D image generated by confocal fluorescence microscopy enabled visualization of the 3D structure of colonic crypts (Figure 3*B*). Optical slices from the 3D reconstructed model showed that mucus in goblet cells and epithelial basement membrane exhibited positive PAFhy staining results (Figure 3*B*). Image processing enabled the highlighting of a single crypt in the 3D reconstructed model (Figure 3*C*), which was straight; mucus in goblet cells was arranged regularly (Supplementary Video 1). In addition, neutrophils exhibited positive PAFhy staining results; this was confirmed by immunohistochemistry using an anti-myeloperoxidase antibody (Figure 3*D*). Positive PAFhy staining findings were not observed when the oxidation step was omitted (Figure 3*E*), suggesting that the principle of PAFhy staining in 3D imaging is similar to the principle in two-dimensional (2D) imaging (Figure 2*D*). The signal obtained after whole-mount staining with Alexa Fluor 488 hydrazide was weaker than the signal obtained after PAFhy staining, unlike 2D imaging (Figure 3*F*). Therefore, we confirmed the establishment of a 3D histopathological system for the volumetric analysis of colonic crypts, which we named PAFhy-3D.

**Figure 4:**
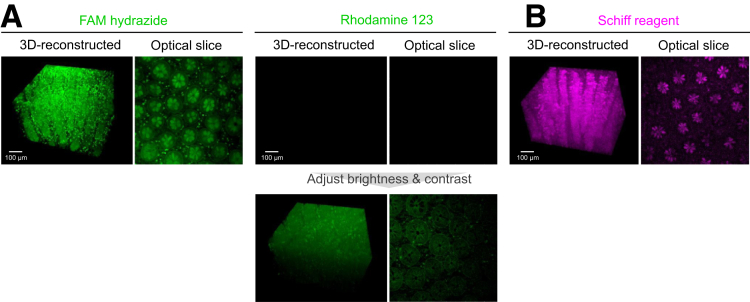
**Comparison of PAFhy and other staining methods.** (*A*) Comparison of PAFhy and rhodamine 123 staining. Human colonic mucosa specimens were stained with FAM hydrazide (PAFhy stain) or rhodamine 123 and subjected to 3D imaging with CUBIC. Under identical staining and imaging conditions, the rhodamine 123 fluorescence signal was significantly weaker than the PAFhy staining result. Although crypt structure was vague if brightness and contrast were increased in 3D reconstruction, the image quality was significantly inferior to the PAFhy staining result. (*B*) Three-dimensional imaging using the Schiff reagent. The Schiff reagent produces weak fluorescence (maximum excitation peak is 540–545 nm and maximum emission peak is 645–650 nm). Indeed, 3D imaging of animal organs is possible using Schiff reagent fluorescence. Therefore, we performed whole-mount staining with the Schiff reagent to 3D imaging of a human colonic mucosa specimen by CUBIC. Although mucus in goblet cells was unevenly stained and crypt structure was partially visible, basal membrane and neutrophils were not imaged. Because the fluorescence property of the Schiff reagent differs from the fluorescence property of FAM hydrazide, strict quantitative comparison with PAFhy staining findings could not be performed. Therefore, the image was obtained and reconstructed under optimal conditions. Scale bar, 100 μm.

Recently, a research group reported a new staining method based on the binding of rhodamine-123 to aldehyde groups produced by the oxidation of periodic acid; they performed clear 3D imaging of infected *Medicago truncatula* roots.^39^ However, the fluorescence signal was significantly weaker than the signal of PAFhy staining used for 3D imaging of CUBIC-cleared human colonic mucosae (Figure 4*A*). Although other 3D Schiff reagent imaging methods in animal organs have been reported,^40^^,^^41^ the resulting image qualities were inferior to the quality of PAFhy staining images (Figure 4*B*).

### Histopathological Examination of Crypts in Ulcerative Colitis

**Figure 5:**
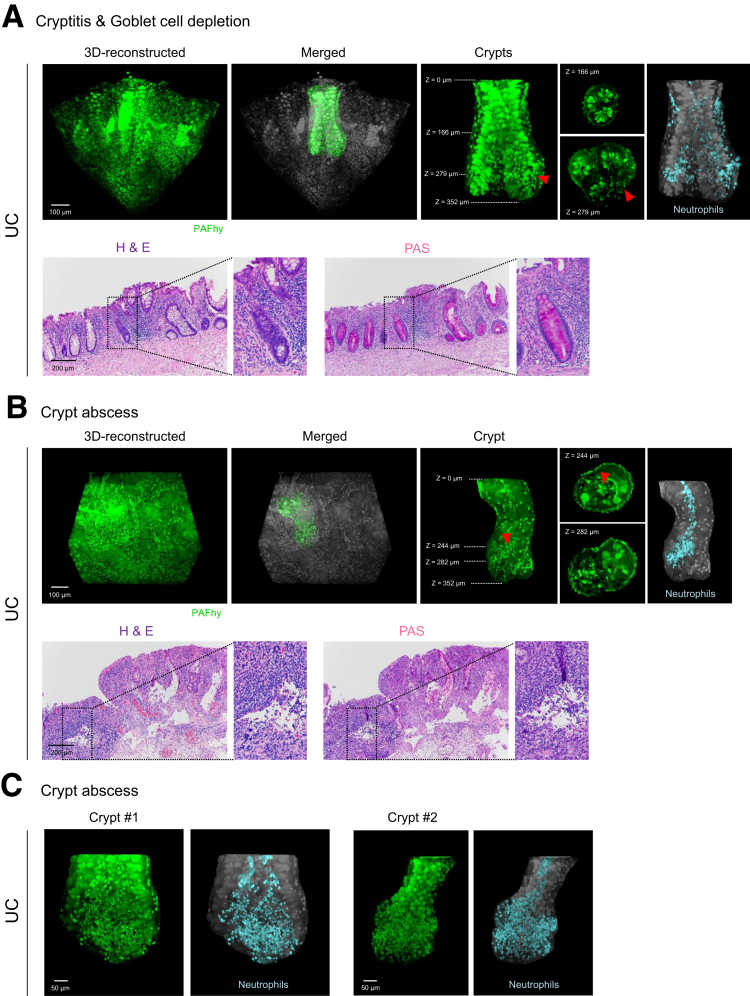
**Histopathological examination by PAFhy-3D of crypts in ulcerative colitis (UC) with severe inflammation.** (*A*) Representative reconstructed 3D images of UC mucosa with cryptitis. Representative doublet crypts with neutrophil infiltration are highlighted and merged with the grayscale background tissue. Optical slices are also presented. Neutrophils in the crypts are highlighted (*light blue*) on the basis of PAFhy-positive findings and cell size in the 3D reconstructed model. Almost all neutrophils around the crypts are highlighted. H&E- or PAS-stained images of the corresponding glass slides are also shown. Scale bar, 100 μm. (*B*) Representative reconstructed 3D images of UC mucosa with crypt abscesses. A representative crypt with micro-abscess is highlighted and merged with the grayscale background tissue. Optical slices are also shown. Neutrophils in the crypt are highlighted (*light blue*) on the basis of PAFhy-positive findings and cell size in the 3D reconstructed model. Only neutrophils continuous with the micro-abscess site are highlighted. H&E- or PAS-stained images from the corresponding glass slides are also presented. Scale bar, 100 μm. (*C*) Other representative reconstructed 3D images of crypt abscesses. Scale bar, 50 μm.

Ulcerative colitis (UC) is a type of IBD for which histopathological characteristics include inflammatory and architectural changes involving crypts (eg, cryptitis, crypt abscess, and crypt distortion). We performed PAFhy-3D on tissue specimens from a UC case and then generated standard H&E- and PAS-stained glass slides from adjacent tissues. The 3D reconstructed images clearly showed cryptitis (Figure 5*A*), in which neutrophils infiltrated the centers of crypts; sites of severe inflammation showed depletion of colonic goblet cells and mucins (ie, goblet cell depletion identified in classical histopathology). Pseudocolor labeling of neutrophils based on PAFhy-positive findings and cell size enabled evaluation of the 3D neutrophil distribution. There were multiple clusters of neutrophils; moreover, neutrophils tended to infiltrate at the bottoms of crypts rather than near the surfaces (Supplementary Video 2). Although lamina propria in the corresponding H&E- and PAS-stained glass slides exhibited moderate to severe neutrophil infiltration, no typical cryptitis was identified (Figure 5*A*).

In another specimen, the crypt lumina were filled with neutrophils (and thus were defined as the crypt abscesses of classical histopathology), and parts of the epithelium were disrupted (Figure 5*B*). Pseudocolor labeling showed that neutrophils invaded diagonally upward within crypts; neutrophil accumulation was greatest at the bottoms of crypts, followed by migration upward in the crypt lumen (Supplementary Video 3). The corresponding H&E- and PAS-stained glass slides showed severe inflammation involving crypts; however, no typical crypt abscesses were found, possibly because of severe destructive changes within the crypts. Other crypts also showed marked neutrophil accumulation, predominantly at the bottom; borders with surrounding stroma were unclear (Figure 5*C*). These findings indicate that PAFhy-3D imaging enables the visualization of a whole crypt in UC, which cannot be performed via conventional 2D histopathology.

### Diagnostic System for Inflammatory Bowel Diseases Based on 3D Crypt Architecture

Next, we verified the utility of PAFhy-3D for clinicopathological examination in IBD. IBDs show characteristic pathological features such as cryptitis, crypt abscess in UC, and epithelioid granuloma in Crohn’s disease (CD).^42^^,^^43^ However, their pathological features are often unclear in random sections, leading to a diagnosis of non-specific colitis (NSC), particularly in mucosae with mild inflammation.^44^ In addition, although crypt distortion is an important characteristic of UC, it is difficult to accurately evaluate the degree of crypt distortion from 2D images on glass slides generated in random positions and directions.

**Figure 6:**
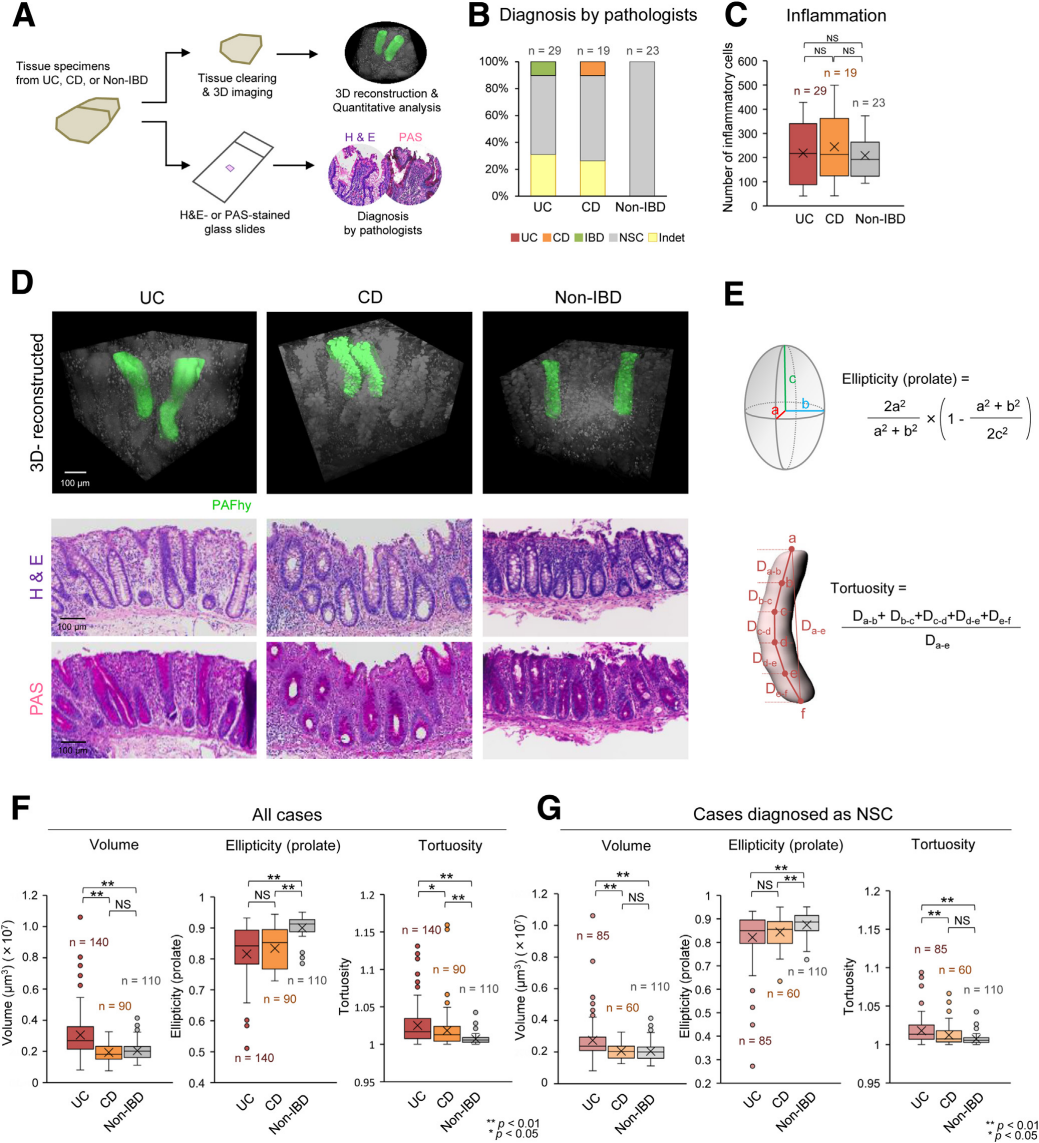
**A diagnostic system for inflammatory bowel diseases (IBDs) based on 3D crypt architecture.** (*A*) Quantitative evaluation of 3D crypt architecture. Colorectal mucosa specimens were randomly sampled from formaldehyde-fixed surgical specimens of UC, Crohn’s disease (CD), or non-IBD (non-tumor area in colorectal cancer specimens) tissues and subjected to PAFhy-3D imaging or histologic evaluation by H&E and PAS staining. (*B*) Seven expert pathologists classified the H&E- or PAS-stained mucosae as UC, CD, IBD, or non-specific colitis (NSC). Twenty-nine specimens of UC (including the specimen in Figure 5), 19 specimens of CD, and 23 specimens of non-IBD were used. Overall, 10.34% of UC specimens were classified as IBD; 10.53% of CD specimens were classified as CD; and 58.62% of UC, 63.16% of CD, and 100% of non-IBD specimens were classified as NSC. For these specimens, 5 or more pathologists were in agreement in terms of classification. Furthermore, 31.03% of UC specimens and 26.32% of CD specimens were classified as indeterminate (Indet). For these specimens, fewer than 5 pathologists were in agreement. (*C*) Histologic evaluation of degree of inflammation in colorectal mucosa specimens. Inflammatory cells per high-power field were counted in 3 random areas of the lamina propria using H&E-stained specimens. NS, not significant. (*D*) Representative 3D reconstructed PAFhy-3D images of UC, CD, and non-IBD specimens. H&E- or PAS-stained images of the corresponding glass slides are also presented. Scale bar, 100 μm (3D reconstructed models) or 200 μm (H&E- and PAS-stained images). (*E*) Illustrations and formulas of definition of ellipticity (prolate) and tortuosity. (*F*) Statistical analysis of volume, ellipticity (prolate), and tortuosity in UC, CD, and non-IBD specimens. Five random crypts were selected from 28 UC, 18 CD, and 22 non-IBD specimens; the quantitative values were calculated using the UC (n = 140), CD (n = 90), and non-IBD (n = 110) crypts. One each of UC, CD, and non-IBD specimens was excluded from the PAFhy-3D analysis because their crypts were almost completely disrupted by inflammation. (*G*) Statistical analysis of volume, ellipticity (prolate), and tortuosity of specimens classified as NSC (*light gray* in [*B*]). Similar to (*F*), 5 random crypts were selected from 17 UC, 12 CD, and 22 non-IBD specimens; the quantitative values were calculated using the UC (n = 85), CD (n = 60), or non-IBD (n = 110) crypts. Statistical analyses were performed by the Mann–Whitney *U* test. ∗∗*P* < 0.01; ∗*P* < 0.05; NS, not significant.

Therefore, we hypothesized that quantitative evaluation of 3D architectural changes in crypts by PAFhy-3D might enable accurate histopathological diagnosis of IBDs. To evaluate this hypothesis, we performed volumetric imaging analysis by PAFhy-3D and 2D classical pathology examination of UC, CD, and non-IBD (ie, mucosae sampled from non-tumor areas of colorectal cancer specimens) tissues. Small pieces of mucosa were randomly sampled from UC, CD, or non-IBD surgical specimens and then cut in half. One half was subjected to volumetric imaging by PAFhy-3D; the other half was subjected to paraffin embedding, sectioning, and H&E and PAS staining (Figure 6*A*). Seven expert pathologists blinded to the clinical data examined the glass slides and classified the mucosal tissues as UC, CD, IBD, or NSC. Overall, 58.62% of UC, 63.16% of CD, and 100% of non-IBD mucosa tissues were classified as NSC (Figure 6*B*). The pathologists classified 31.03% of UC and 26.32% of CD specimens as indeterminate. No specimen was correctly classified as UC, and only 10.53% were correctly classified as CD, reflecting the difficulty in diagnosing IBDs. The degree of inflammation was not significantly different among the specimens (Figure 6*C*).

PAFhy-3D imaging enabled clear visualization of distorted crypts in UC and CD specimens, which were not necessarily obvious in the corresponding H&E- or PAS-stained glass slides (Figure 6*D*). For quantitative analysis, we evaluated volume, ellipticity, and tortuosity (Figure 6*E*) on the basis of the 3D data. Among all specimens, the crypt volumes were significantly larger in UC than in CD and non-IBD specimens (Figure 6*F*). The crypt ellipticity value was significantly smaller in UC and CD than in non-IBD specimens. Crypt tortuosity was greatest in UC, followed by CD and non-IBD specimens. When the specimens classified as NSC (Figure 6*B*) were used, significant differences were observed in volume and ellipticity (Figure 6*G*). Crypt tortuosity in UC was significantly greater than in CD and non-IBD specimens; however, it did not significantly differ between CD and non-IBD specimens. Thus, the quantitative 3D values were significantly different among UC, CD, and non-IBD specimens, but they could not be distinguished by the pathologists. Therefore, quantitative evaluation based on PAFhy-3D enables accurate clinicopathological diagnosis.

**Figure 7:**
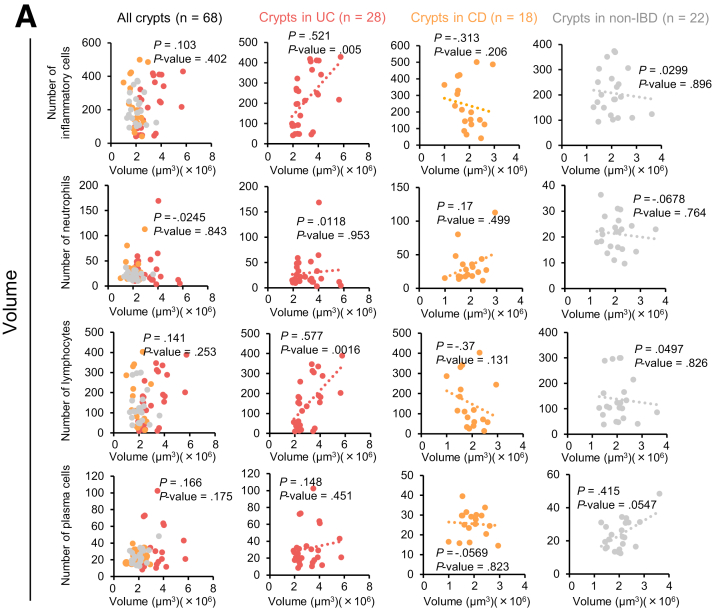

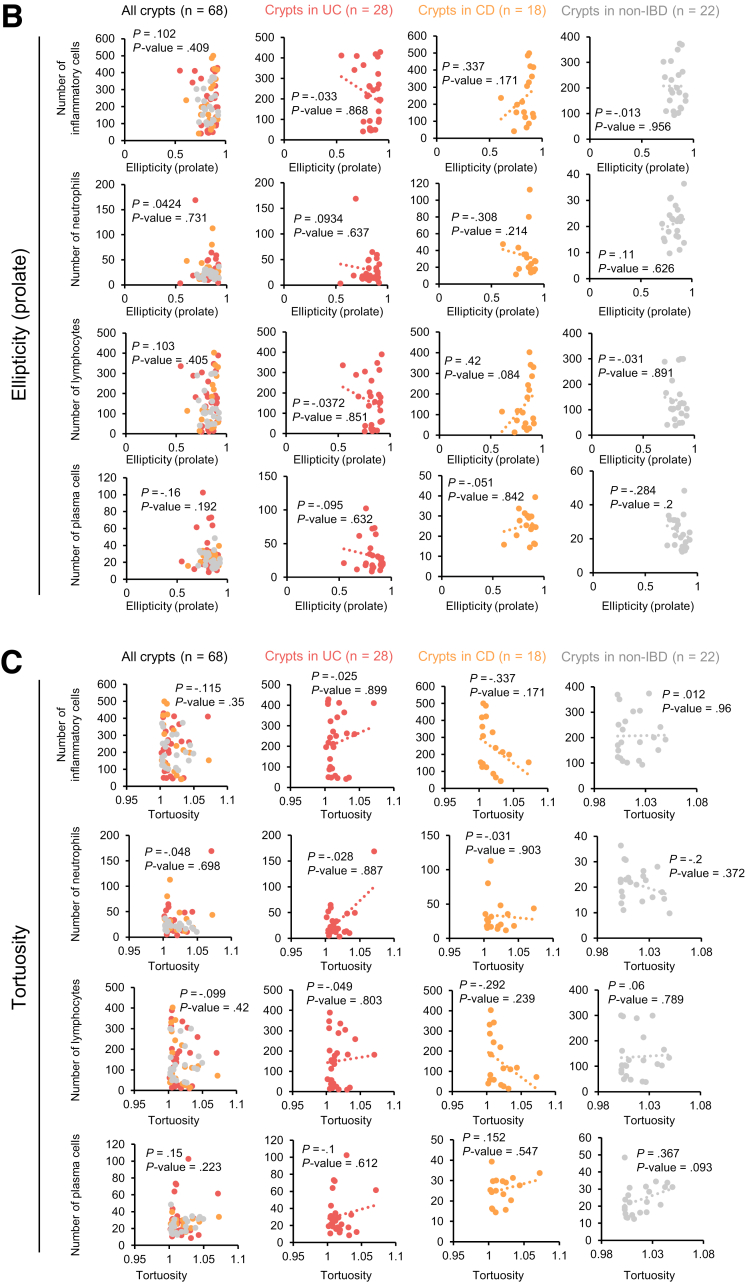
**Correlation between crypt volume, ellipticity, or tortuosity and the number of infiltrating inflammatory cells.** The numbers of infiltrating total inflammatory cells, neutrophils, lymphocytes, and plasma cells per high-power field were counted in 3 random areas of the lamina propria on the H&E-stained glass slides used in Figure 6*C*. The mean numbers of each type of inflammatory cell were calculated for all specimens. The average crypt volume (*A*), ellipticity (*B*), or tortuosity (*C*) of each specimen were calculated using the data from Figure 6*F*. Correlations between the averaged 3 quantitative values and the number of each type of infiltrating inflammatory cell were evaluated; Spearman rank correlation coefficients were calculated using EZR software.

We explored whether the 3 quantitative parameters were correlated with the numbers of infiltrating total inflammatory cells, neutrophils, lymphocytes, or plasma cells. The crypt volumes in the UC specimens were positively correlated with the numbers of total inflammatory cells and lymphocytes (Figure 7). A weak inverse correlation between the crypt volume and number of total inflammatory cells or lymphocytes in CD patients was found, although the differences did not attain statistical significance (Figure 7*A*). Ellipticity seemed to be associated with the number of lymphocytes in CD samples, but statistical significance was not achieved (Figure 7*B*). Tortuosity exhibited a weak negative correlation with the total number of inflammatory cells in CD samples but a weak positive correlation with the number of plasma cells in NSC samples (Figure 7*C*). Again, the differences did not attain statistical significance.

### Three-Dimensional Histopathological Examination of Distorted Crypts in Inflammatory Bowel Diseases

**Figure 8:**
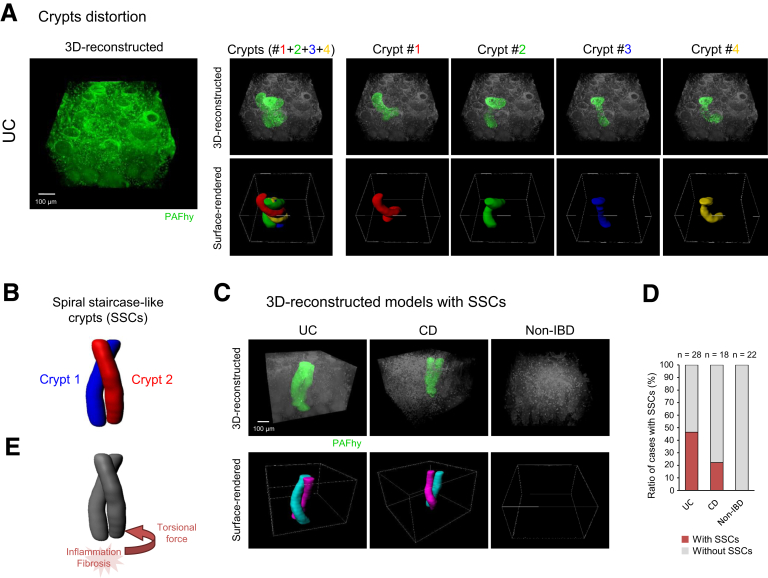
**PAFhy-3D histopathological examination of distorted crypts in IBDs.** (*A*) Representative 3D reconstructed image with the most severe crypt distortion. Four crypts with counterclockwise distortion are independently highlighted. Scale bar, 100 μm. (*B*) Definition of spiral staircase-like crypts (SSCs). SSCs consist of 2 or more crypts with the same twisting direction. (*C*) Representative 3D reconstructed images of UC (n = 28) or CD (n = 18) with SSCs. SSCs were not observed in non-IBD specimens (n = 22). Scale bar, 100 μm. (*D*) Rates of specimens with SSCs; 46.43% of UC specimens and 22.22% of CD specimens had SSCs. (*E*) Mechanism of SSC formation. Two or more adjacent crypts might be regionally distorted by a torsional force generated by focal inflammation and/or fibrosis, with the resulting increase in pressure.

A 3D image of UC with the most severe crypt distortion was examined (Figure 8*A*). In this specimen, up to 4 crypts were involved in the same lesion; all crypts were twisted counterclockwise (Supplementary Video 4). In other specimens, a portion of the mucosa also contained 2 or more crypts twisting in the same direction. We defined this finding as spiral staircase-like crypts (Figure 8*B*). Spiral staircase-like crypts were found in UC and CD but not in non-IBD specimens (Figure 8*C*). The rates of spiral staircase-like crypts were 46.43% in UC and 22.22% in CD (Figure 8*D*). This finding might explain crypt twisting, in which 2 or more adjacent crypts are regionally distorted by a torsional force generated by focal inflammation and/or fibrosis, with the resulting increase in pressure (Figure 8*E*).

### Application of PAFhy-3D to a Deep Learning-Based Diagnostic System

**Figure 9:**
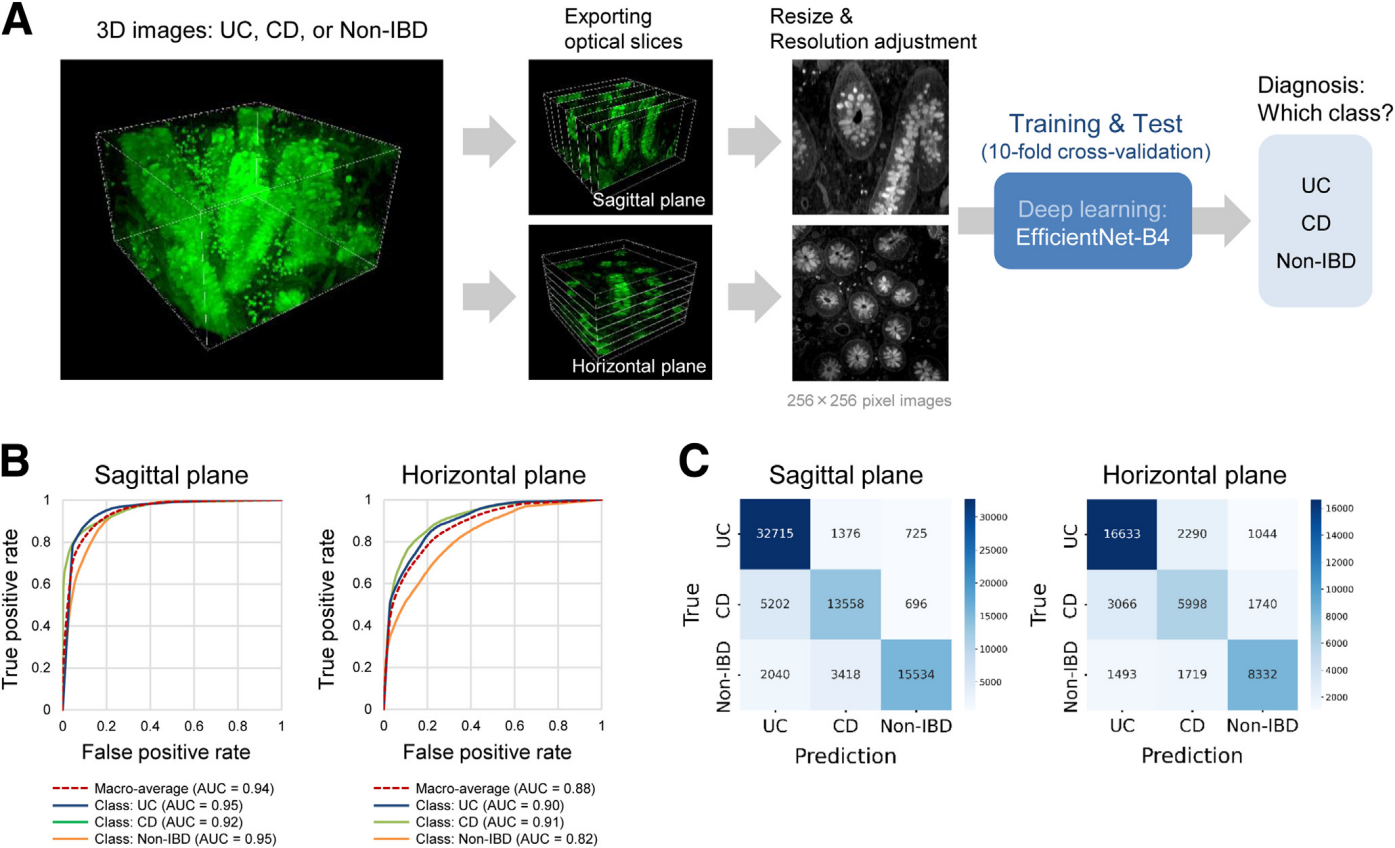
**Deep learning-based diagnostic system.** (*A*) Overview of convolutional neural networks for development of deep learning system (DLS) for differential diagnosis of IBDs. Sagittal or horizontal optical slice images were exported from 3D images of UC (n = 27), CD (n = 17), or non-IBD (n = 19) specimens obtained by PAFhy-3D and then converted to grayscale 256 × 256 pixel images. Subsequently, these images were subjected to training and testing by EfficientNet-B4. Data were used as described in Figure 6; however, 2 UC, 2 CD, and 4 non-IBD specimens were excluded because of poor image quality. (*B*) Receiver operating characteristic curves for differential diagnosis of UC, CD, or non-IBD. (*C*) Confusion matrices for differential diagnosis of IBDs by DLS using sagittal or horizontal plane images.

**Figure 10:**
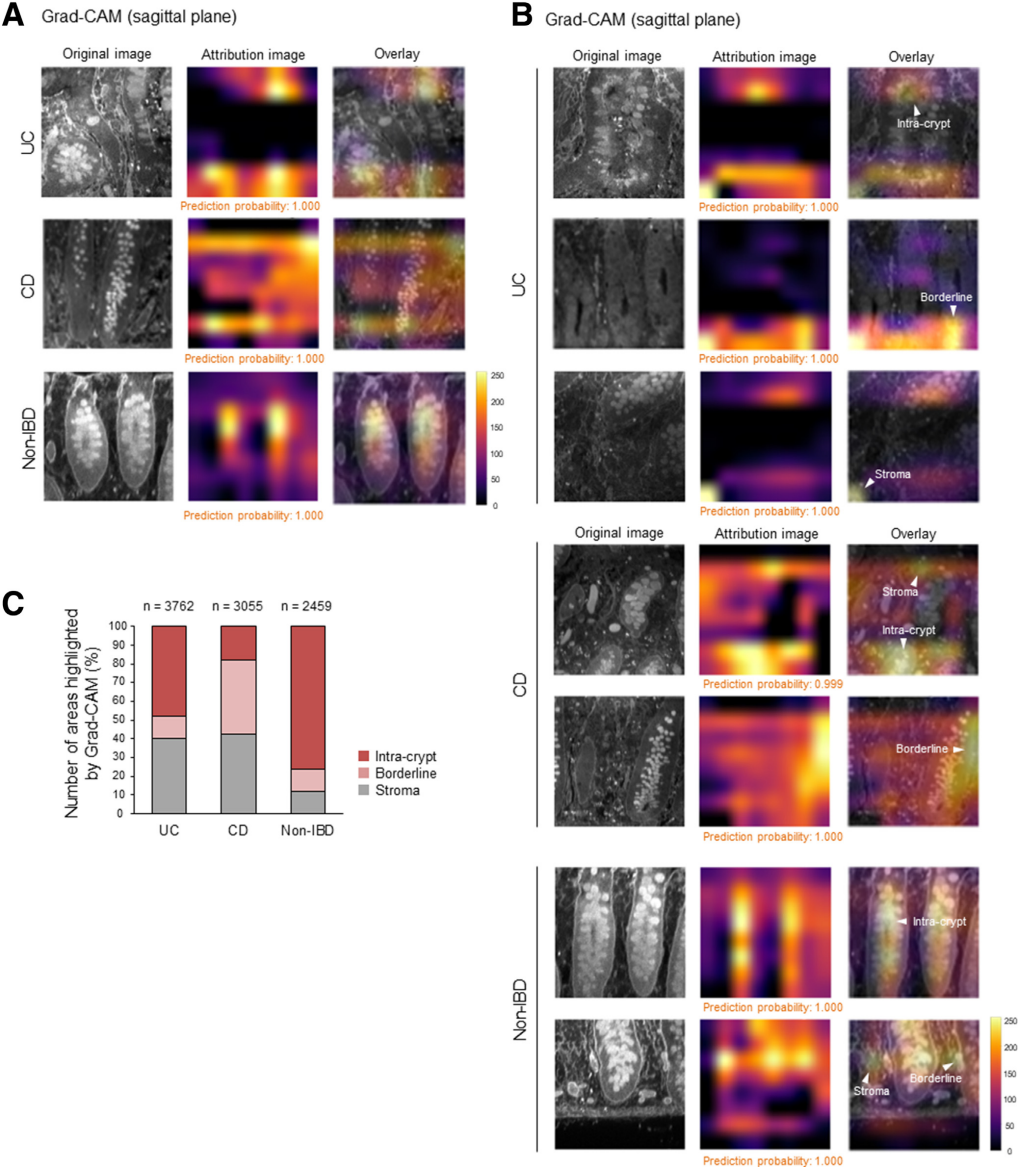
**Visualization by Grad-CAM.** (*A*) Representative images of UC, CD, and non-IBD visualized by gradient-weighted class activation mapping (Grad-CAM). (*B*) Other representative images of UC, CD, or non-IBD specimens visualized by Grad-CAM, in which intra-crypt, borderline between crypt and stroma, and extra-crypt stromal areas are highlighted. (*C*) Classification of areas on which the neural network focused. All areas highlighted by Grad-CAM in sagittal plane images derived from 3D data of 2 UC (3762 areas), 2 CD (3055 areas), or 2 non-IBD (2459 areas) specimens with especially good prediction probabilities were counted and classified. In UC images, the areas on which the DLS focused consisted of 47.95% of intra-crypt areas, 11.75% of borderline areas between crypts and stroma, and 40.3% of extra-crypt stromal areas. In CD images, the areas on which the DLS focused consisted of 18.17% of intra-crypt areas, 39.38% of borderline areas, and 42.46% of stromal areas. In non-IBD images, the areas on which the DLS focused consisted of 76.05% of intra-crypt areas, 12.04% of borderline areas, and 11.92% of stromal areas.

We next applied PAFhy-3D to a diagnostic system based on a convolutional neural network architecture, EfficientNet-B4^45^ (Figure 9*A*). Sagittal or horizontal optical slice images were exported from the 3D images and subjected to stratified-group 10-fold cross-validation. Strikingly, the deep learning system (DLS) enabled differential diagnosis of these diseases using images obtained by PAFhy-3D. Using sagittal plane images, the DLS achieved a macro-averaged area under the curve (AUC) of 0.94 (AUC of 0.95 for UC, 0.92 for CD, and 0.95 for non-IBD). In contrast, using horizontal plane images, the DLS achieved AUCs of 0.88, 0.90, 0.91, and 0.82, respectively (Figure 9*B*). On the basis of sagittal plane images, the DLS achieved an accuracy of 87.59% and F1 score of 0.875 for classification of UC, accuracy of 85.79% and F1 score of 0.717 for classification of CD, and accuracy of 90.86% and F1 score of 0.819 for classification of non-IBD (Figure 9*C*, Table 1). Analysis of these predictions using Grad-CAM^46^ indicated that the DLS focused on the intra-crypt region and borderline areas between crypts and stroma, as well as extra-crypt stroma, particularly for UC and CD (Figure 10).

**Table 1:** Performance of Deep Learning System in Terms of Differential Diagnosis of Inflammatory Bowel Diseases Using Sagittal Plane Images Derived From Periodic Acid and Fluorescein Dye FAM Hydrazide-3D Data

	Sagittal plane		
	UC	CD	Non-IBD
Accuracy (*%*)	87.59	85.79	90.86
Sensitivity (*%*)	93.97	69.69	74.00
Specificity (*%*)	82.10	91.41	97.38
Precision (*%*)	81.88	73.88	91.62
F1 score	0.875	0.717	0.819

CD, Crohn’s disease; IBD, inflammatory bowel disease; UC, ulcerative colitis.

### PAFhy-3D Imaging of Other Diseases

**Figure 11:**
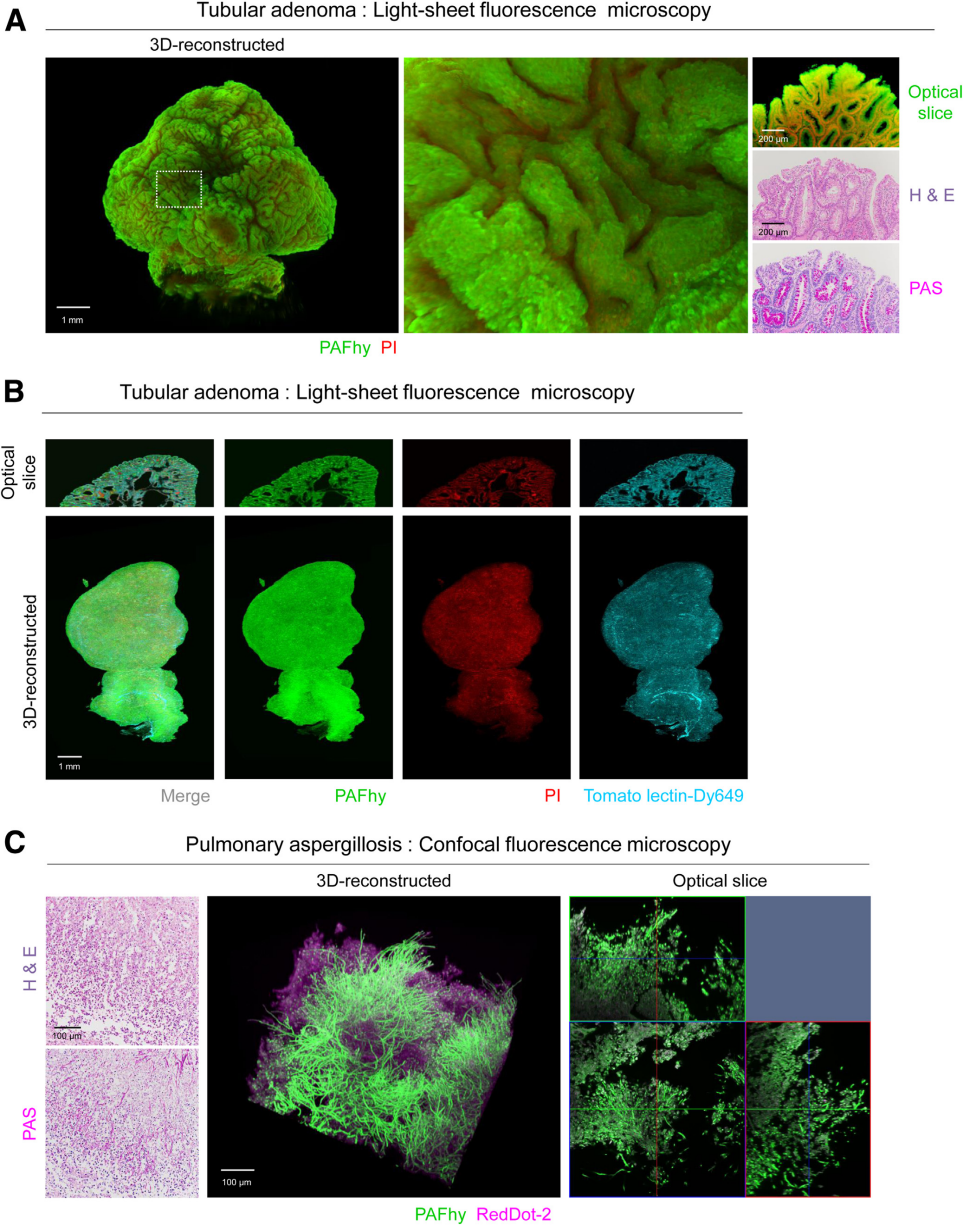
**PAFhy-3D imaging for other diseases.** (*A*) Reconstructed 3D image of whole colonic polyp obtained by light-sheet fluorescence microscopy. An optical slice image derived from this 3D model is also shown. This polyp was subjected to generation of H&E- and PAS-stained glass slides after PAFhy-3D imaging and subsequent phosphate-buffered saline washes. H&E- and PAS-stained histopathological images show that the polyp consists of tubular adenoma. Scale bar, 1 mm (3D model) or 200 μm (optical slice, H&E, PAS). (*B*) PAFhy-3D imaging of a human colonic polyp by light-sheet fluorescence microscopy combined with vascular structure staining by a fluorescence-conjugated lectin. A colonic polyp was cleared with CUBIC reagents and stained with FAM hydrazide, propidium iodide (PI), and DyLight 649-conjugated Lycopersicon esculentum (Tomato) lectin and then imaged by light-sheet fluorescence microscopy. Only this 3D image underwent imaging processing with a normalized filer.^36^ The images of individual channels (Ch-PAFhy with an excitation wavelength at 488 nm, Ch-PI at 592 nm, and Ch-Tomato lectin-Dy649 at 642 nm) were independently downscaled into 1/15.4 (from 2048 × 2048 to 133 × 133 pixel images). These images were subjected to image processing with a normalized filer. Subsequently, a mask image (pixels of >200 signal intensity were regarded as positive) was generated from the Ch-PAFhy image. This mask image was applied to images of Ch-PI and Ch-Tomato lectin-DyLight 649. Scale bar, 1 mm. (*C*) Reconstructed 3D image of lung tissue with pulmonary aspergillosis. H&E- and PAS-stained images from glass slides generated from the same lesion and optical-slice images of orthographic projection derived from the 3D model are also shown. Scale bar, 100 μm.

Finally, we applied PAFhy-3D imaging to diseases other than IBDs. PAFhy-3D imaging of a colonic polyp of tubular adenoma by light-sheet fluorescence microscopy showed protuberances on the polyp surface (Figure 11*A*). Fluorescence-conjugated lectin was used to label vascular structures, which were imaged by PAFhy-3D (Figure 11*B*). In addition, *Aspergillus* hyphae in a pulmonary aspergillosis specimen, which exhibit positive PAS staining results, were visualized by PAFhy-3D (Figure 11*C,* Supplementary Video 5). A 3D reconstructed model generated by confocal fluorescence microscopy showed hyphae branching structures.

## Discussion

In this study, we developed PAFhy staining and the 3D imaging technique PAFhy-3D. PAFhy staining is based on an oxidative process where polysaccharides react with periodic acid to produce an aldehyde, which is bound by FAM hydrazide (Figure 2*D*). The PAFhy-3D imaging method enabled visualization of structures with a high carbohydrate proportion, including mucus in goblet cells, basal membrane, and neutrophils in colonic crypts. Thus, PAFhy staining has utility for 3D pathology similar to PAS staining.

We examined the 3D structure of crypts in colonic mucosae by means of PAFhy-3D. PAFhy signals in the basal membrane and mucus in goblet cells enabled the examination of 3D crypt architecture (Figure 3). Using this method, we identified 3 important components in tissue. First, we found multiple sites in which a large number of neutrophils infiltrated cryptitis lesions in UC (Figure 5*A*). Second, we observed micro-abscesses in UC predominantly at the crypt bottom (ie, the crypt abscesses of classical histopathology); these were spatially continuous with neutrophils that infiltrated diagonally inward and upward (Figure 5*B*). Third, we identified multiple distorted crypts in the same UC lesion, all of which were twisted counterclockwise (Figure 8*A*). In addition, we found spiral staircase-like crypts in IBD mucosae; these were more frequent in UC than in CD (Figure 8*C* and *D*). An area containing adjacent multiple crypts might be distorted by the torsional force that arises from the pressure difference induced by inflammation and/or fibrosis (Figure 8*E*). Such structural changes may accumulate because of repeated instances of inflammation during the active and resolving phases of UC.^42^ PAFhy-3D imaging enables the visualization of morphology and histopathology. Further studies based on PAFhy-3D will elucidate pathological mechanisms and identify novel disease-specific pathological findings.

Important findings in classical histopathology analysis of UC include crypt distortion, cryptitis, and/or crypt abscesses.^42^ However, accurate evaluation of crypt distortion is difficult using 2D images that are generated in random positions and directions. Indeed, in this study, pathologists did not correctly classify most UC and CD specimens (Figure 6*B*). Therefore, we hypothesized that quantitative evaluation of crypt distortion by 3D imaging might enhance the histopathological diagnosis of IBDs. As expected, crypt volume, ellipticity, and tortuosity significantly differed among UC, CD, and non-IBD specimens (Figure 6*F* and *G*). Such differences were also evident in specimens that pathologists classified as NSC. Therefore, PAFhy-3D quantitative evaluation enables differential diagnosis of IBDs. Mucosa specimens that contain crypts of increased volume and decreased ellipticity should be classified as UC; specimens with crypts of normal volume and decreased ellipticity should be classified as CD. In addition, 3-stage quantitative evaluation based on crypt tortuosity could contribute to the diagnosis of IBDs. Comparison of these 3 quantitative parameters with the numbers of infiltrating total inflammatory cells, neutrophils, lymphocytes, and plasma cells revealed positive correlations between the crypt volume and number of total inflammatory cells or lymphocytes in the UC specimens but negative and weak correlations in the CD specimens (Figure 7*A*). Thus, UC and CD tissues may differ in terms of the direction of crypt volume changes in response to chronic inflammation.

Furthermore, the image data obtained by PAFhy-3D were used for DLS (Figure 9). The DLS achieved excellent performance (macro-averaged AUC = 0.94; F1 score = 0.875, 0.717, and 0.819 for UC, CD, and non-IBD, respectively) for differential diagnosis of IBDs; this identification could not be performed by pathologists. Notably, DLS performance was greater when sagittal rather than horizontal plane images were used for training and testing (Figure 9*B*). This supports the hypothesis that DLS diagnosis is based on 3D crypt architecture, particularly distortion, because such 3D morphologic changes are more evident in sagittal view. Indeed, among the areas on which the DLS focused, approximately 60% in UC and CD images and approximately 90% in non-IBD images were intra-crypt or borderline areas between crypts and stroma (Figure 10). However, approximately 40% of the areas in UC and CD and approximately 10% of the areas in non-IBD images were extra-crypt stroma, suggesting that the findings on which the DLS focused are not necessarily limited to morphologic changes in crypts.

PAFhy staining and PAFhy-3D imaging enabled 3D histopathological analysis in greater depth and enhanced the accuracy of clinicopathological diagnosis. Thus, these techniques have potential for use in experimental and clinical pathology.

## Methods

### Clinical Specimens

The tissue specimens used for screening fluorescent probes and the specimen exhibiting pulmonary aspergillosis were from patients who underwent pathological dissection at Osaka University Hospital in 2017. The colorectal mucosa specimens used for 3D imaging were from patients who underwent surgery at Osaka University Hospital in 2020 or 2021. For quantitative analysis, 29 UC tissue specimens were sampled from 6 surgical specimens, 19 CD tissues were sampled from 5 surgical specimens, and 23 non-IBD tissues were sampled from 5 surgical specimens. The tissue specimens were sampled from surgical specimens in independent and distant areas of different colors. The study was approved by the Ethical Review Board of the Graduate School of Medicine, Osaka University (approval nos. 14470 and 18187).

### Fluorescence Staining of Tissue Sections

Human colonic mucosa tissue was washed with phosphate-buffered saline (PBS), immersed in 30% (w/v) sucrose in PBS, and frozen in O.C.T. compound (45833; Sakura Finetek, Torrance, CA) at −80°C overnight. Frozen sections were cut at a thickness of 10 μm using a cryostat (CM3050S; Leica Biosystems, Wetzlar, Germany). The frozen sections were washed 3 times with PBS and then pretreated with 0.5% periodic acid (HIO_4_) solution (86171; Muto Pure Chemicals, Tokyo, Japan) at room temperature for 30 minutes (Figure 2*B*). Sections were washed 3 times with PBS and then incubated with FAM hydrazide, 5-isomer (50 μmol/L, BP-23934; BroadPharm, San Diego, CA), Alexa Fluor488 hydrazide (50 μmol/L, A10436; Thermo Fisher Scientific, Waltham, MA), BDP FL hydrazide (50 μmol/L, 11470; Lumiprobe, Hunt Valley, MD), or fluorescein (50 μmol/L, F0095; Tokyo Chemical Industry, Tokyo, Japan) in PBS at room temperature overnight. After additional PBS washes, the tissue sections were mounted with Fluoro-KEEPER antifade reagent with DAPI (12745-74; Nacalai Tesque, Kyoto, Japan) and imaged by confocal fluorescence microscopy.

### Tissue Clearing and PAFhy Staining

CUBIC-L and CUBIC-R+ reagents were used for tissue clearing (Tokyo Chemical Industry; T3740 and T3741). Tissue clearing was performed in accordance with the standard protocol shown in Figure 3*A*. For colonic polyp specimens that were cleared and imaged with light-sheet fluorescence microscopy, the durations of CUBIC-L treatment, staining, and CUBIC-R+ treatment were extended by 1 day. Briefly, formaldehyde-fixed tissue specimens were washed with PBS, immersed in 50% (v/v) CUBIC-L reagent (1:1 mixture of water: CUBIC-L) overnight, and immersed in CUBIC-L reagent for 5 days (Figure 3*A*) or 6 days (Figure 11*A* and *B*) at 37°C with gentle shaking. The specimens were next washed with PBS, immersed in 30% (w/v) sucrose in PBS, and frozen in O.C.T. compound at −80°C overnight. The frozen specimens were thawed, washed with PBS, and immersed in 0.5% periodic acid solution (86171; Muto Pure Chemicals) at room temperature for 30 minutes. After additional PBS washes, the specimens were stained with FAM hydrazide, 5-isomer (50 μmol/L, BP-23934; BroadPharm) in PBS with 0.5% (v/v) Triton X-100 (12967; Nacalai Tesque) at room temperature for 2 days (Figure 3*A*) or 3 days (Figure 11*A* and *B*) with gentle shaking. If nuclear counterstaining was needed, propidium iodide (10 μg/mL, P21493; Life Technologies, Carlsbad, CA) or RedDot-2 (1:100, 40061-T; Biotium, Fremont, CA) was added to the staining solution. If vascular structure staining was needed, DyLight 649-conjugated Lycopersicon esculentum (Tomato) lectin (1:50, DL-1178; Vector Laboratories, Burlingame, CA) was added to the staining solution. After staining had been performed, the specimens were washed with PBS, immersed in 50% (v/v) CUBIC-R+ reagent (1:1 mixture of water: CUBIC-R+) overnight, and immersed in CUBIC-R+ reagent for 1 day (Figure 3*A*) or 2 days (Figure 11*A* and *B*) with gentle shaking. The cleared tissue specimens were subjected to 3D imaging by confocal fluorescence (Figure 3, Figure 4, Figure 5, Figure 6, 8, 9, and 11*C*) or light-sheet fluorescence microscopy (Figure 11*A* and *B*).

### Whole-Mount 3D Immunohistochemistry

Whole-mount immunohistochemistry with CUBIC tissue clearing was conducted as previously described.^15^ A tissue specimen was treated with 50% (v/v) CUBIC-L reagent overnight and CUBIC-L reagent for 5 days, washed with PBS, immersed in 30% (w/v) sucrose in PBS, and frozen in O.C.T. compound at −80°C overnight. The frozen specimen was thawed, washed with PBS, and subjected to PAFhy staining. After the specimen had been washed with PBS, it was immersed in Blocker Casein in PBS (37528; Thermo Fisher Scientific) at room temperature for 2 hours. The specimen was then washed with PBS and subjected to immunostaining with 1:50 diluted Alexa Fluor 647-conjugated anti-myeloperoxidase antibody (ab252131; Abcam, Cambridge, UK) in 0.5% (v/v) Triton X-100 in PBS at room temperature for 2 days. The specimen was washed with PBS at room temperature and cross-linked in 1% paraformaldehyde in PBS for 1 hour at room temperature. The stained specimen was immersed in 50% CUBIC-R+ reagent overnight and CUBIC-R+ reagent for 1 day. Images of the lamina propria surface were obtained by confocal microscopy.

### Microscopy and Image Analysis

Three-dimensional images of human colonic mucosae and lung tissue with pulmonary aspergillosis shown in Figure 3, Figure 4, Figure 5, Figure 6, 8, 9, and 11*C* were acquired using a confocal fluorescence microscope (LSM880 Confocal/Multiphoton; Carl Zeiss, Oberkochen, Germany). A 3D image of a colonic polyp shown in Figure 11*A* was acquired using a light-sheet fluorescence microscope (Lightsheet 7; Carl Zeiss). A 3D image of a colonic polyp shown in Figure 11*B* was acquired using a custom-designed light-sheet microscope.^36^ Raw image data were reconstructed and analyzed with Imaris software (version 9.2.1; Bitplane, Zurich, Switzerland). Crypt highlighting was performed by 3D rendering with Imaris. Crypt area was determined on the basis of PAFhy-positive basal membrane at 13-μm (Figures 3, 5, 8 or 66-μm (Figure 6) optical slice intervals, followed by automatic 3D reconstruction and surface rendering. Imaris was also used to calculate the 3 parameters defined in Figure 6. Volume was calculated by a preset statistic in Imaris. Ellipticity (prolate) was also preset in the statistics of Imaris and calculated using the formula shown in Figure 6*E*. Tortuosity was semi-manually calculated. In each crypt at 250 μm depth, a point indicating the crypt center was marked in each 50-μm optical slice (Figure 6*E*). Distances between points (D_a-b_, D_b-c_, D_c-d_, D_d-e_, and D_e-f_) were measured by Imaris, as was the shortest distance between the top and bottom of the crypt (D_a-f_). Tortuosity was calculated as follows: (D_a-b_+D_b-c_+D_c-d_+D_d-e_+D_e-f_)/(D_a-f_). To normalize the analyses, the 3 quantitative parameters were calculated using data from crypts located no deeper than 250 μm from the mucosal surfaces, even if the specimens were thicker than that value.

### Deep Learning System

The DLS was based on EfficientNet-B4,^45^ a state-of-the-art convolutional neural network architecture, with TensorFlow 2.5^47^ (Figure 9*A*). Optical slice images exported from 3D images of UC, CD, or non-IBD specimens were used for DLS training and testing. Stratified-group 10-fold cross-validations were performed to evaluate DLS performance.

### Data Preparation for the Deep Learning–Based Diagnostic System

Three-dimensional data of 63 specimens (27 UC, 17 CD, and 19 non-IBD) were used to train and evaluate the DLS (Figure 9). These data were same as the data used in Figure 6; however, 3D data with poor image quality (2 UC, 2 CD, and 4 non-IBD) were excluded from the analysis. Sagittal and horizontal plane images were extracted from the 3D data using AICSImageIO^48^ and preprocessed as follows. Because of inconsistent slice size, each sagittal plane image was cropped to a square from the center at the maximum possible size. Subsequently, the cropped images were resized to 256 × 256 pixels. Horizontal plane images were 512 × 512 pixels; they were resized to 256 × 256 pixels. Finally, the pixel values were rescaled to a range of 0 to 1 via division by 255.

### Construction of the Deep Learning System

The DLS was based on EfficientNet-B4^45^ and used Grad-CAM^46^ to produce visual explanations for the predictions. The input and output shapes of EfficientNet-B4 were changed to 256 × 256 and 3, respectively; the weights were randomly initialized. Adam and the categorical cross-entropy were used as the optimizer and loss function. The epochs, learning rate, and batch size hyperparameters were set to 30, 0.001, and 16, respectively; early stopping with the patience of 10 epochs was used to prevent overfitting. A learning rate schedule that used exponential decay with a decay rate of 0.95 was applied to the Adam optimizer at each epoch after 5 epochs. While training the models, data-augmentation techniques based on horizontal and vertical flips, random rotation between –20° and 20°, and random erasing^49^ were used to improve prediction performance. To evaluate the DLS, a stratified-group 10-fold cross-validation was performed. All models were trained using TensorFlow version 2.5.0 and an NVIDIA GeForce RTX 3090 with 24 GB of memory.

### Evaluation Metrics

To evaluate the diagnostic performance of the DLS, we evaluated its accuracy, sensitivity, specificity, precision, and F1 score. The F1 score is the harmonic mean of precision and recall calculated using the following equation: F1 score = 2 precision recall/(precision + recall).

### Statistical Analyses

Statistical analysis was performed using Microsoft Excel (Redmond, CA) or EZR.^50^ The Shapiro–Wilk test was used to evaluate the normality of the data distribution at a significance level of .05. If normality was confirmed, the homogeneity of variance was determined by F test at a significance level of 0.05. If 2 groups were normally distributed with or without equal variance, Student *t* test or Welch *t* test was applied, respectively. When the normality of data could not be assumed, the Mann–Whitney *U* test was used. Spearman rank correlation coefficients were calculated using EZR software.

## Supplementary Material

Suppementary Video 1

Suppementary Video 2

Suppementary Video 3

Suppementary Video 4

Suppementary Video 5
